# Cysteine hydropersulfide reduces lipid peroxidation and protects against myocardial ischaemia-reperfusion injury - Are endogenous persulfides mediators of ischaemic preconditioning?

**DOI:** 10.1016/j.redox.2023.102605

**Published:** 2023-01-10

**Authors:** Kayleigh Griffiths, Tomoaki Ida, Masanobu Morita, Reece J. Lamb, Jordan J. Lee, Michael P. Frenneaux, Jon M. Fukuto, Takaaki Akaike, Martin Feelisch, Melanie Madhani

**Affiliations:** a Institute for Cardiovascular Sciences, College of Medical and Dental Sciences, University of Birmingham, Birmingham, UK; b Department of Environmental Medicine and Molecular Toxicology, Tohoku University Graduate School of Medicine, Sendai, 980-8575, Japan; c Academic Health System, Hamad Medical Corporation, Doha, Qatar; d Department of Chemistry, Sonoma State University, California, USA; e Clinical and Experimental Sciences, Faculty of Medicine, University of Southampton, Southampton, UK

**Keywords:** Cysteine persulfide, Hydrogen sulfide, Ischaemia-reperfusion injury, Lipid peroxidation, Hypoxia, Oxidative stress

## Abstract

Earlier studies revealed the presence of cysteine persulfide (CysSSH) and related polysulfide species in various mammalian tissues. CysSSH has both antioxidant and oxidant properties, modulates redox-dependent signal transduction and has been shown to mitigate oxidative stress. However, its functional relevance in the setting of myocardial ischaemia-reperfusion injury (IRI) remains unknown. The present study was undertaken to (1) study the dynamics of production and consumption of persulfides under normoxic and hypoxic conditions in the heart, and (2) determine whether exogenous administration of the CysSSH donor, cysteine trisulfide (Cys-SSS-Cys) at the onset of reperfusion rescues functional impairment and myocardial damage by interfering with lipid peroxidation. Utilising a well-established *ex vivo* Langendorff murine model, we here demonstrate that endogenous tissue concentrations of CysSSH are upregulated when oxygen supply is compromised (global myocardial ischaemia) and rapidly restored to baseline levels upon reperfusion, suggestive of active regulation. In a separate set of experiments, exogenous administration of Cys-SSS-Cys for 10 min at the onset of reperfusion was found to decrease malondialdehyde (MDA) concentrations, formation of 4-hydroxynonenal (4-HNE) protein adducts and rescue the heart from injury. Cys-SSS-Cys also restored post-ischaemic cardiac function, improving both coronary flow and left ventricular developed pressure (LVDP). Taken together, these results support the notion that endogenous CysSSH plays an important role as a “redox preconditioning” agent to combat the oxidative insult in myocardial IRI.

## Graphical abstract

**Figure:**
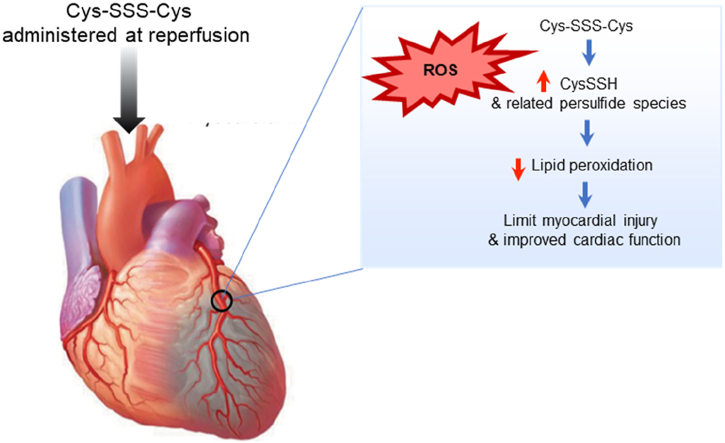

## 1 Introduction

Much of cellular redox signaling relies on the exchange of electrons within a dense and highly dynamic network of chemical interactions between inorganic reactive sulfur species, small aminothiols and protein sulfhydryls, enriched by ample cross-talk with other oxygen- and nitrogen-based reactive species [1,2]. In the last decade, hydrogen sulfide (H_2_S) based therapeutics have evolved to treat cardiovascular disease [3,4], but their molecular mode of action remains ill defined. Hydropersulfides (RSSH), specifically cysteine persulfide (CysSSH) and related sulfane-sulfur species such as glutathione persulfide (GSSH) and disulfane (HSSH), have recently emerged as endogenous and surprisingly abundant cell/tissue constituents that may explain some of the biological effects previously attributed to H_2_S [5]. Essentially based on primordial origin-of-Life chemistry, this research field is still in its infancy [6]. In part due to its short half-life and the associated analytical challenges the biological functions of CysSSH remain incompletely understood. Its endogenous formation likely involves multiple pathways, including enzymes of the transulfuration pathway, cysteinyl-tRNA synthetase, CARS2 (which is tightly linked to mitochondrial function [7]) and non-enzymatic reaction routes, depending on cell type, tissue and organism [6,[8], [9], [10]]. Investigating the role of CysSSH *in vivo* remains a challenging task because polysulfur species are taken up by cells and subject to complex metabolism [11,12], complicating the interpretation of experimental results. Most studies carried out until now have therefore employed simple *in vitro* systems. Yet, despite impressive cell-protective activities of hydropersulfides, few pharmacological and/or interventional mechanistic studies have been carried out to date in isolated organ systems or *in vivo* using precursors that give rise to RSSH [13]. This is particularly true for cardiovascular research, with one notable exception: a very recent study using isolated perfused hearts and cultured cardiomyocytes demonstrated that hydropersulfides outperform other reactive sulfur species (in particular reduced glutathione) in limiting IRI, possibly by virtue of their ability to preserve mitochondrial respiration [14].

Acute myocardial infarction (AMI) remains the leading cause of morbidity and mortality worldwide [15]. Currently, prompt restoration of blood flow with percutaneous coronary intervention remains the treatment of choice for reducing myocardial injury. Paradoxically, as a consequence of this procedure, restoration of blood flow to the ischaemic zone leads to apoptosis and necrosis resulting to IRI [16,17]. Increased reactive oxygen species (ROS) production has been shown to disrupt redox signaling in the early phase of reperfusion. A shift in the balance between oxidants and antioxidants in favour of oxidants promotes oxidative stress and cellular damage by various mechanisms, such as the peroxidation of membrane lipids [18]. The products of lipid oxidation include reactive aldehydes, such as 4-hydroxynonenal (4-HNE) and malondialdehyde (MDA), and there is accumulating evidence to show that increased levels of both 4-HNE and MDA are formed during myocardial IRI [19].

Cysteine trisulfide (Cys-SSS-Cys; also known as thiocystine) is a stable reservoir of sulfane sulfur that can serve as donor/precursor of hydropersulfides by rapidly reacting with sulfide and other thiols to generate CysSSH and related sulfane-sulfur polysulfide species [20]. Previous studies in *E.coli* bacteria and cultured cells demonstrated that treatment with Cys-SSS-Cys generates CysSSH, conferring antioxidant protection against electrophile-induced cell death [10,21]. Recent studies revealed that enhancement of persulfides by synthetic persulfide donors promotes anti-inflammatory effects in macrophages [22] and inhibits lipid peroxidation in cultured fibroblasts [23]. Whilst these findings have unravelled some unique properties of persulfides *in vitro*, the physiological relevance of CysSSH in tissue/organs remains unclear. While a very recent investigation demonstrated that hydropersulfides (persulfides) can inhibit lipid peroxidation by scavenging reactive aldehydes such as 4-HNE and MDA in cultured cells [24], whether such effects also occur in an intact organ system remains to be shown. Another important knowledge gap relates to changes in concentration of CysSSH and related polysulfide species in the target tissue of the relevant experimental model. As a proof-of-concept study, we here show that the concentration of endogenous CysSSH and related polysulfides dynamically changes during ischaemia and reperfusion of the heart as oxygen supply of the tissue is altered. Additionally, utilising heart homogenate *in vitro* and an isolated perfused mouse model of myocardial IRI, we demonstrate that Cys-SSS-Cys results in rapid generation of CysSSH, and that exogenous administration of this persulfide donor at the onset of reperfusion is associated with reduced lipid peroxidation-derived reactive aldehyde formation and improved cardiac function.

## 2 Material and methods

### 2.1 Chemicals and reagents

Cys-SSS-Cys and SSP4 were synthesised as previously described [10,25] and used as provided. All other chemicals were of the highest purity commercially available and obtained either from Merck, Fisher Scientific or VWR, unless specified elsewhere.

### 2.2 Ex vivo Langendorff perfusion of the heart

**Fig. 1:**
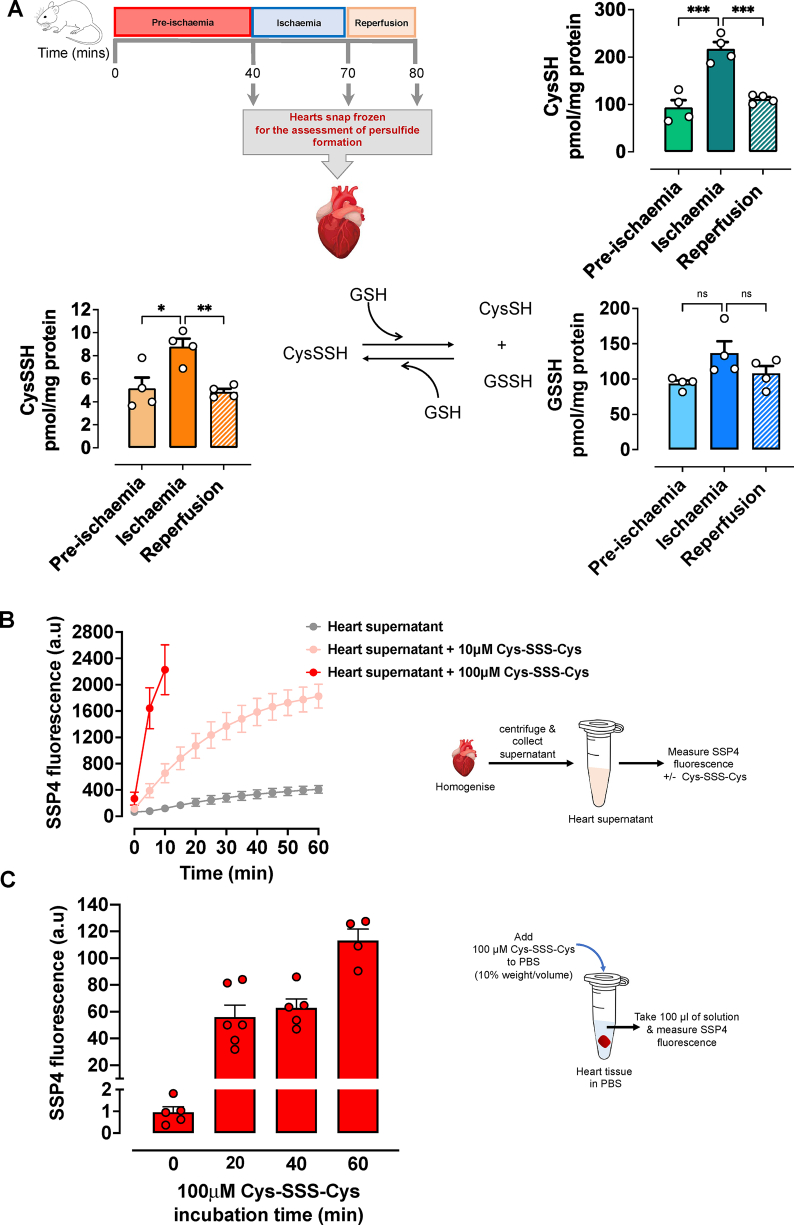
Persulfides are produced endogenously during myocardial ischaemia. **(A)** Isolated, retrogradely perfused C57BL/6 mouse hearts were subjected to a well-established ischaemia/reperfusion protocol. Hearts were snap frozen either after 40 min of stabilisation (pre-ischaemia), 30 min of global ischaemia, or global ischaemia followed by 10 min of reperfusion. Frozen tissue was analyzed for the endogenous thiols and persulfides cysteine (CysSH), cysteine persulfide (CysSSH) and glutathione persulfide (GSSH) by liquid chromatography-electrospray ionization tandem mass spectrometry (LC-ESI-MS/MS; n = 4 per group). Concentrations of endogenous CysSH and CysSSH were markedly elevated following ischaemia when compared to pre-ischaemia. Data are expressed as means ± SEM; *P ≤ 0.05, **P ≤ 0.01, ***P ≤ 0.001 as determined by one-way ANOVA with Bonferroni's test. **(B)** Persulfides are generated during metabolism of cysteine trisulfide. Time-dependent increases in fluorescence of the sulfane sulfur specific probe, SSP4 from cardiac supernatants incubated with either control vehicle or cysteine trisulfide (Cys-SSS-Cys; 10 or 100 μM) was measured over a period of 10 or 60 min. Arbitrary units (a.u). **(C)** Persulfide leakage from cardiac tissue. Time-dependent leakage of persulfides from cardiac tissue was measured via fluorescence response to SSP4 at 20, 40 and 60 min following treatment with 100 μM Cys-SSS-Cys. Data expressed as mean ± SEM (n = 4–6 group; see methods for details).

**Fig. 2:**
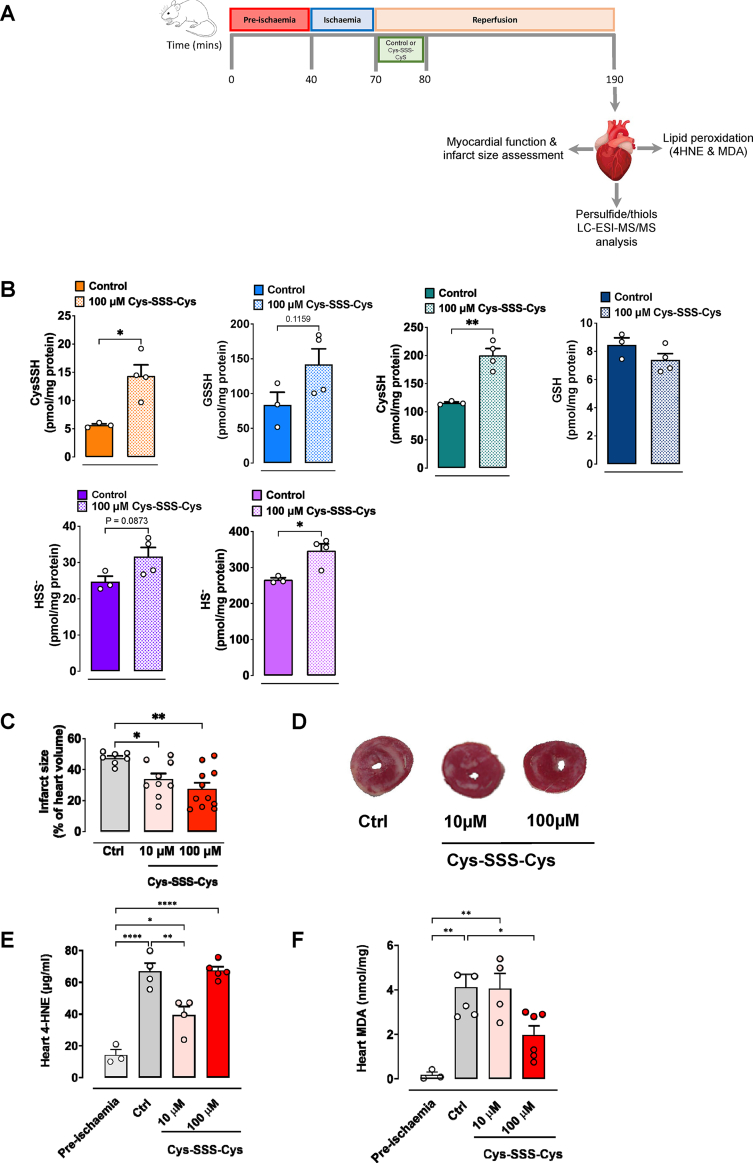
Cysteine trisulfide generates persulfides and attenuates myocardial injury and lipid peroxidation. **(A)** Experimental protocol implemented to assess persulfide formation, infarct size and lipid peroxidation following treatment of isolated perfused hearts with either control vehicle (Krebs) or cysteine trisulfide (Cys-SSS-Cys). In all experiments, C57BL/6 mouse hearts were subjected to 40 min of stabilisation, 30 min global ischaemia and 2 h of reperfusion. At the onset of reperfusion, hearts were perfused with control (KHB) or Cys-SSS-Cys (10 or 100 μM) for the first 10 min, followed by perfusion with Krebs. **(B)** Utilising LC-ESI-MS/MS analysis the following persulfides/thiols were measured in heart homogenates: cysteine persulfide (CysSSH), glutathione persulfide (GSSH), cysteine (CysSH), glutathione (GSH), hydrosulfide (HS^-^), and inorganic disulfide (HSS^-^). Cys-SSS-Cys significantly increased CysSH, CysSSH and HS^-^ levels in the isolated hearts when administered at the onset of reperfusion (n = 3–4 per group). Data are presented as mean ± SEM, *P ≤ 0.05, **P ≤ 0.01 vs control, as determined by unpaired *t*-test. **(C)** Cys-SSS-Cys significantly reduced cardiac infarct size in a concentration-dependant manner (n = 7–11 per group). Data expressed as mean ± SEM, *P ≤ 0.05, **P ≤ 0.01, as determined by one-way ANOVA with Bonferroni's test. **(D)** Representative images of TTC stained heart sections, treated with vehicle control, 10 μM or 100 μM Cys-SSS-Cys. Red staining indicates viable tissue and white areas indicate non-viable (necrotic) tissue. **(E, F)** The extent of myocardial lipid peroxide formation was assessed by measurement of 4-hydroxynonenal (4-HNE) protein adducts (n = 3–4 per group) and malondialdehyde (MDA; n = 3–6 per group) concentrations in hearts subjected to 40 min stabilisation (pre-ischaemia) or myocardial I/R injury in hearts perfused with either control vehicle (Krebs) or Cys-SSS-Cys (10 and 100 μM). Data displayed are means ± SEM; *P ≤ 0.05, **P ≤ 0.01, ****P ≤ 0.0001 as determined by one-way ANOVA with Bonferroni's test. (For interpretation of the references to color in this figure legend, the reader is referred to the Web version of this article).

All experiments were carried out in accordance with UK (Scientific Procedures) Act of 1986 and EU Directive 2010/63/EU. Male C57BL/6 mice (25–30 g; Charles Rivers Laboratories) were anticoagulated (0.1 IU sodium heparin) and anesthetized with sodium pentobarbital (300 mg/kg i.p.). Following anaesthesia, excised hearts were mounted onto a Langendorff apparatus and retrogradely perfused at a constant pressure of 80 mmHg with Krebs Henseleit buffer (KHB; mM:118.5 NaCl, 25.0 NaHCO_3_, 4.75 KCl, 1.18 KH2PO_4_, 1.19 MgSO_4_, 11.0 glucose, and 1.4 CaCl_2_, equilibrated with 95% O_2_ and 5% CO_2_ at 37 °C [26,27]. Atrial pacing was performed at 600 beats/min and coronary flow was measured via the STH Pump Controller (ADInstruments). To assess left ventricular developed pressure (LVDP) and left ventricular end-diastolic pressure (LVEDP), a fluid-filled balloon was inserted into the left ventricular chamber. Coronary flow, LVDP and LVEDP were recorded using Labchart 7 (ADInstruments). After 40 min of stabilisation, isolated hearts were subjected to 30 min global ischaemia and reperfusion (10 min or 2 h, Fig. 1, Fig. 2, respectively) [26]. At reperfusion, hearts were perfused with either vehicle (KHB) or Cys-SSS-Cys (10 μM or 100 μM in KHB) for 10 min, with KHB given for the remaining reperfusion period. Two minutes into ischaemia, electrical pacing was stopped and restarted 5 min into reperfusion. Upon completion of the 2 h reperfusion, hearts were assessed for infarct size, as a proportion of area at risk (AAR), by planimetry using Image J software [26]. Identical Langendorff experiments were carried out for the assessment of cardiac lipid peroxidation (MDA and 4-HNE) and endogenous persulfide formation, respectively.

### 2.3 Measurement of lipid peroxidation

The extent of lipid peroxides generated following myocardial IRI was assessed by measuring the levels of 4-HNE protein adducts using a competitive ELISA (Abcam 238538) and MDA by colorimetric assay (modified thiobarbituric acid-reactive substances assay; Abcam ab118970).

### 2.4 Determination of persulfide formation

Persulfide formation was assessed employing two complementary methods: (1) qualitative monitoring using the sulfane-sulfur specific fluorescent probe SSP4 [25] and (2) quantitation of defined species by high performance liquid chromatography/electrospray ionization tandem mass spectrometry (LC-ESI-MS/MS) [7,28]. For the former, excised hearts were kept frozen at −80 °C before homogenization in ice-cold RIPA buffer (1:10 w/v); incubations with SSP4 (10 μM) were carried out at 37 °C in the presence and absence of Cys-SSS-Cys (or cysteine/cystine as negative controls) while recording fluorescence changes (emission 482 nm, excitation 514 nm) every 10 min for up to 1 h. To assess persulfide efflux, frozen tissue was cut into pieces, suspended in RIPA buffer, cleared of debris and incubated in Falcon tubes at 37 °C in the absence/presence of Cys-SSS-Cys; supernatant aliquots of these incubations were collected at defined intervals and reacted with SSP4 for 10 min before recording final fluorescence intensities. For the mass spectrometry-based experiments, hearts were homogenized with 5 mM HPE-IAM in 70% ice-cold methanol/30 mM sodium acetate buffer (pH 6.5) and incubated at 37 °C for 20 min. Homogenates were centrifuged at 15,000×*g* for 10 min, and the supernatants were diluted with 0.1% formic acid containing known amounts of isotope-labelled internal standards. The samples were then analyzed by LC-ESI-MS/MS to determine cysteine (CysSH), CysSSH, glutathione (GSH), GSSH, and inorganic persulfide (HSSH; disulfane) as HPE-IAM adducts, respectively. The precipitate obtained by centrifugation following methanol extraction was dissolved in PBS containing 0.1% SDS, and protein quantification was performed by the BCA method.

### 2.5 Statistical analysis

All data are presented as means ± standard error of the mean (SEM), where *n* is the number of animals. Statistical analysis was conducted by GraphPad Prism software (version 9.0) using unpaired *t*-test, one-way ANOVA and two-way ANOVA for comparisons, with Bonferroni's correction being used for multiple group comparisons, P ≤ 0.05 was considered significant.

## 3 Results and discussion

We here demonstrate that endogenous CysSSH levels increase during myocardial ischaemia, that exogenous application of Cys-SSS-Cys at the onset of reperfusion rescues the heart from I/R damage and associated impairment of cardiac function, and that these effects are associated with reduced lipid peroxidation.

There is increasing evidence in the literature to show *in vivo* biosynthesis of sulfane sulfur-containing species [29], yet the pathophysiological relevance of CysSSH in the cardiovascular system remains unknown. Therefore, in the present study we first sought to establish the presence and metabolic profile of endogenous CysSSH and related persulfide species (CysSH and GSSH) in the heart following normoxia (pre-ischaemia), hypoxia (ischaemia), and 10 min of reperfusion (Fig. 1A). Using LC-MS/MS analysis, we here demonstrate for the first time that intracellular CysSSH concentrations significantly increase following myocardial ischaemia compared with pre-ischaemic mouse hearts (Fig. 1A). A similar profile was observed for cysteine (CysSH) itself. The magnitude of these alterations within such a short period of time was unexpected. One possible explanation for our findings is that the steady-state levels of both, intracellular CysSH and CysSSH are augmented as a result of reduced cysteine dioxygenase activity (which, under aerobic conditions, oxidizes cysteine to cysteinesulfinic acid to produce taurine and sulfate) and impaired oxidative H_2_S metabolism by sulfide-quinone oxidoreductase (SQR) during myocardial ischaemia [[30], [31], [32]]. Those alterations are consistent with the involvement of H_2_S in oxygen sensing [33] and *in vivo* observations in acclimatized humans documenting robust elevation of circulating cysteine concentrations in the hypobaric hypoxia of high altitude compared to normoxia at sea-level [34]. Whilst GSSH followed a similar trend as CysSSH in the heart, those changes did not reach statistical significance. Remarkably, within 10 min of reperfusion, steady-state levels of tissue CysSSH, CysSH and GSSH had all returned back to pre-ischaemic levels, suggestive of a relatively short half-life and/or increased consumption during reperfusion. While it remains unknown whether longer periods of reperfusion could further lower tissue CysSSH levels, it is remarkable that steady-state concentrations achieved after 10 min of reperfusion were almost identical to pre-ischemic levels, suggestive of active regulation. The observed profile of tissue metabolite changes during ischaemia/reperfusion further suggests that CysSSH may underpin, at least in part, the protection afforded by ischemic preconditioning. Given its potential physiological significance this hypothesis would seem to merit further investigation.

Previous *in vitro* studies have shown that treatment of HEK293 cells with Cys-SSS-Cys (0.2–1 mM) generates CysSSH and GSSH [10]. Given that CysSSH levels were reduced upon reperfusion, we next sought to confirm that exogenous administration of Cys-SSS-Cys leads to an elevation of Cys-SSH concentration in cardiac tissue. This was first investigated in feasibility studies using the sulfane-sulfur specific fluorimetric probe, SSP4. Addition of Cys-SSS-Cys to murine cardiac homogenate revealed rapid and concentration-dependent elevation of tissue persulfide levels (Fig. 1B). The gradual fluorescence increase observed with cardiac homogenates in the absence of Cys-SSS-Cys suggests hydropersulfides to be endogenously formed already under basal conditions. Additional experiments carried out using pieces of intact heart tissue documented the release of intracellularly formed CysSSH into the extracellular space (Fig. 1C). The latter suggests CysSSH egress from the heart, at least after pharmacological elevation of endogenous concentrations with Cys-SSS-Cys. To test whether those results can be translated to the setting of myocardial I/R injury, we next measured the concentration of endogenously formed CysSSH and related thiol/polysulfide species in vehicle and Cys-SSS-Cys (100 μM) treated hearts (Fig. 2A). Administration of Cys-SSS-Cys (100 μM) within the first 10 min of the reperfusion period led to robust elevation of intracellular levels of CysSSH, CysSH, HS^-^ and HSS^-^ when compared to control hearts perfused with vehicle only (Fig. 2B). GSSH tissue concentrations tended to increase as well, although the elevation of tissue GSSH levels was not significant. In spite of the relatively modest number of independent experimental observations, the results of these experiments demonstrated a pattern similar to that depicted in Fig. 1A. Taken together, these experiments provide convincing experimental evidence that a pharmacological concentration (100 μM) of Cys-SSS-Cys robustly elevates cardiac CysSSH concentrations well above baseline levels.

The fact that those elevated persulfide levels remained detectable at the end of the 2 h reperfusion period suggests that tissue concentrations in the first minutes of reperfusion must have been even higher and should have afforded protection against ROS-induced tissue damage and depressed cardiac function. Indeed this was the case as we observed a significant reduction in infarct size (expressed as the percentage of area at risk) with 10 μM Cys-SSS-Cys (33.8 ± 3.8 *vs.* 47.3 ± 1.5% control-vehicle; P < 0.04), which was further attenuated with 100 μM of Cys-SSS-Cys (27.6 ± 3.9 *vs* 47.3 ± 1.5% control-vehicle; P < 0.002; Fig. 2C). Our results thus corroborate and extend previous findings that demonstrated persulfide-generating precursors to exhibit potent cardioprotective effects in an *ex vivo* [14] and *in vivo* murine model of myocardial IRI [13].

To investigate whether the cardioprotective effects of CysSSH we observed in our model might have been mediated by inhibition of lipid peroxidation, two of the most frequently used markers [19] were measured in cardiac tissue: 4-HNE and MDA. Two complimentary techniques were used: formation and reactivity of 4-HNE was assessed by ELISA using a specific antibody against its protein adducts whereas MDA (and related reactive aldehyde) levels were assessed using a colorimetric assay. As expected, both lipid peroxide markers were increased in isolated hearts perfused with control vehicle and subjected to myocardial IRI when compared with pre-ischaemic hearts (Fig. 2E and F, respectively). Interestingly, hearts treated with lower concentrations (10 μM) of Cys-SSS-Cys showed significantly reduced 4-HNE adduct levels compared to the vehicle control, but this attenuation was lost at higher concentrations of Cys-SSS-Cys (100 μM; Fig. 2E). This U-shaped response may be related to the unique chemistry of CysSSH, which has both, antioxidant and oxidant properties [35]. In contrast, we observed the opposite effect when measuring free MDA levels in cardiac tissue (Fig. 2F), whereby significant reductions in MDA were only seen with 100 μM Cys-SSS-Cys. This may be related to the fact that MDA originates from a multitude of sources and that its endogenous levels are considerably higher compared to those of 4-HNE.

**Fig. 3:**
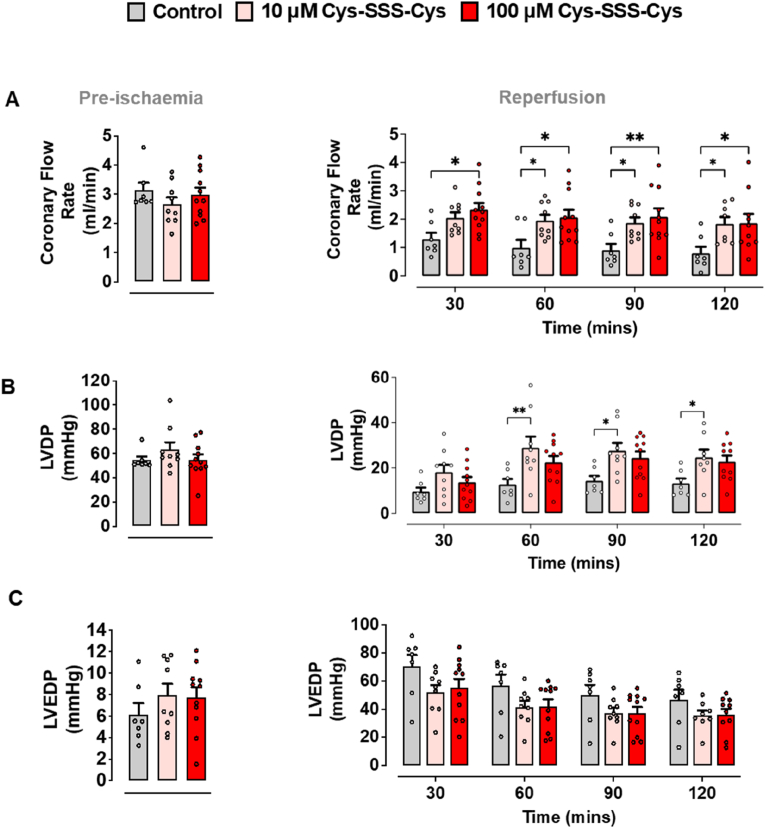
Cysteine trisulfide improves cardiac function following myocardial ischaemia reperfusion. As depicted in Fig. 2, the same Langendorff protocol was used for the assessment of cardiac function and infarct size. **(A)** Coronary blood flow was measured during pre-ischaemia and following 2 h of reperfusion. A fluid-filled ballon was inserted into the left ventricle chamber for the assessment of **(B)** left ventricular diastolic blood pressure (LVDP) and **(C)** left ventricular end-diastolic blood pressure (LVEDP) at pre-ischaemia and 2 h of reperfusion, respectively. Cys-SSS-Cys significantly improved coronary flow and LVDP when compared to control-treated hearts when administered as a post-conditioning agent (n = 7–11 per group). Data expressed as means ± SEM, *P ≤ 0.05, **P ≤ 0.01 (two-way ANOVA with Bonferroni correction).

Functional measurements carried out in parallel with our biochemical experiments further corroborated these results. As in the pharmacological experiments described above, isolated mouse hearts were subjected to 40 min stabilisation (pre-ischaemia), 30 min global ischaemia, and 2 h reperfusion (Fig. 2A). During the stabilisation period (pre-ischaemia), there were no significant differences between treatment groups for baseline coronary flow, LVDP and LVEDP (Fig. 3A–C). Following global ischaemia and reperfusion, control mouse hearts revealed lower coronary flow (0.78 ± 0.23 ml/min), reduced LVDP (13.23 ± 2.13 mmHg), and increased LVEDP (46.65 ± 7.0 mmHg; Fig. 3A–C). Exogenous administration of Cys-SSS-Cys (10 and 100 μM) right at the onset of reperfusion significantly improved cardiac hemodynamic parameters, including coronary flow and contractile function (LVDP; Fig. 3A and B). A similar trend of improvement in LVDP was also observed with the lower Cys-SSS-Cys concentration (10 μM; Fig. 3B), although this was not significantly different at all time points when compared with the control group. An attenuation in LVEDP was observed upon Cys-SSS-Cys treatment, but this was not significant (Fig. 3C). Coronary vascular resistance and myocardial contractility are dynamically regulated by independent processes. Curiously, whereas coronary flow increased or plateaued with higher Cys-SSS-Cys concentrations, LVDP at 30–60 min appeared to reflect the U-shaped response observed for 4-HNE protein adduct formation.

**Fig. 4:**
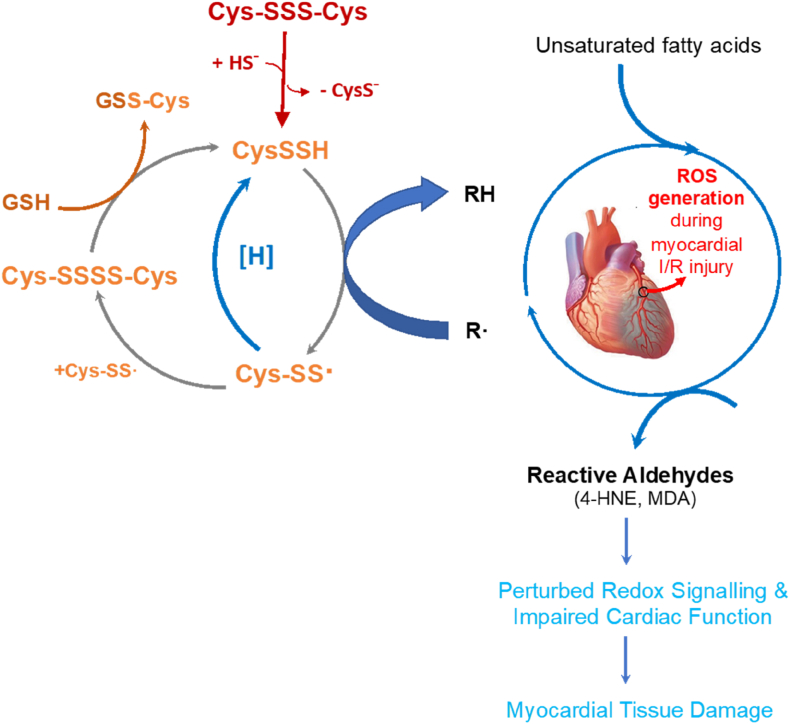
Proposed mechanism underpinning the protective effects of Cys-SSS-Cys in myocardial ischaemia/reperfusion injury. When administered at the onset of reperfusion, Cys-SSS-Cys generates cysteine persulfide (CysSSH), which acts as a chain-breaking antioxidant by trapping radical species generated during I/R. This in turn gives rise to cysteinylpersulfide radical (Cys-SS^.^) formation the dimerization of which produces cysteine tetrasulfide (Cys-SSSS-Cys). One possible fate of RSS^.^ is that it converts back to RSSH. [H] represents biological reduction via enzymatic and/or non-enzymatic reactions akin to what happens with e.g., the ascorbyl radical. Together, these reactions provide an effective mechanism for radical trapping, attenuating lipid peroxidation, limiting myocardial injury and improving cardiac function.

Collectively, our results up to this juncture indicate that the reduction in lipid peroxidation and/or formation of reactive aldehyde protein adducts (which are known to have the potential to perturb redox regulation [18]) following pharmacological elevation of CysSSH levels is associated with reduced myocardial tissue damage and enhanced myocardial functional recovery. However, associations do not imply causation, and a molecular mechanism is required to place these results mechanistically into context. To this end, an elegant and very recent study (although not carried out in heart tissue) may shine light on the mechanism of the cardioprotective effects of CysSSH we here describe. The results of this particular study suggested that hydropersulfides may exert their cytoprotective effects via an autocatalytic regenerative pathway that contributes to their potent inhibitory activity against radical chain reactions that trigger lipid peroxidation [24]. Another recent study revealed an association between lipid peroxidation and ferroptosis [36], which is an alternative process driven by ROS. A hypothetical mechanism that encompasses all of our results is depicted in Fig. 4. Whether or not those reactions involve additional interaction with metals and/or metalloproteins such as myoglobin [37] remains to be investigated. Moreover, ROS-induced oxidation of unsaturated fatty acids gives rise to an array of complex reaction products including lipid peroxides and reactive aldehydes of which only two exemplary products have been investigated in the present study. Reactive aldehydes such as MDA and 4-HNE may be the culprit in the tissue damage, but to demonstrate a clear cause/effect relationship would require much more detailed information about the compartmentalisation of different species within the myocardial tissue and their interaction with distinct subcellular targets. Unfortunately, such fine-grained information is currently neither available for reactive aldehydes nor CysSSH. Nevertheless, a close relationship between the cellular production of CysSSH and mitochondrial bioenergetics has been established [7], and 4-HNE is known to be particularly reactive towards mitochondrial proteins; together, this may explain the curious association between protein adduct formation and recovery from IRI-related contractility impairment in the first half hour following Cys-SSS-Cys administration. In any case, our data support the notion that hydropersulfides are potent protective entities against myocardial IRI, inviting the concept that endogenous CysSSH and related sulfane-sulfur species may play an important role as redox “preconditioning” agents. Thus, cysteine hydropersulfide may play a crucial role in the development of new therapeutic strategies to combat oxidative damage in patients with myocardial IRI.

## Funding

This work was funded by the 10.13039/501100000274British Heart Foundation (PG/19/87/34792) awarded to M.M, M.P.F and MF. Grants-in-Aid for Scientific Research [(S), (A), (B), (C), Challenging Exploratory Research, Transformative Research Areas] from the 10.13039/501100001700Ministry of Education, Culture, Sports, Science and Technology, Japan, to T.A (18H05277, 20K21496 and 21H05263), T.I (20K07306), and M.Mo (19K07341); Japan Science and Technology (S22098), 10.13039/501100003382CREST Grant Number JPMJCR2024, Japan, to T.A; 10.13039/100009619Japan Agency for Medical Research and Development (AMED) Grant Number JP21zf0127001, Japan, to T.A. 10.13039/501100001691Japan Society for the Promotion of Science Invitational Fellowship awarded to M.M (s22098).

## Author contributions

MM conceived the study and wrote the first draft of the manuscript. MM and MF designed experiments, and JMF, MPF, and TA, provided important intellectual content. KG, TI, MMo, RL, and JL conducted experiments, acquired the data and performed analyses. All authors critically reviewed, interpreted the data and approved the final version of the manuscript.

## Declaratio of Competing interest

Authors declare they have no conflict of interests.

## Data Availability

Data will be made available on request.
